# Estimating the lifetime risk of a false positive screening test result

**DOI:** 10.1371/journal.pone.0281153

**Published:** 2023-02-15

**Authors:** Tim White, Sara Algeri

**Affiliations:** 1 Department of Statistics, University of Michigan, Ann Arbor, Michigan, United States of America; 2 School of Statistics, University of Minnesota, Minneapolis, Minnesota, United States of America; Aminu Kano Teaching Hospital, NIGERIA

## Abstract

False positive results in screening tests have potentially severe psychological, medical, and financial consequences for the recipient. However, there have been few efforts to quantify how the risk of a false positive accumulates over time. We seek to fill this gap by estimating the probability that an individual who adheres to the U.S. Preventive Services Task Force (USPSTF) screening guidelines will receive at least one false positive in a lifetime. To do so, we assembled a data set of 116 studies cited by the USPSTF that report the number of true positives, false negatives, true negatives, and false positives for the primary screening procedure for one of five cancers or six sexually transmitted diseases. We use these data to estimate the probability that an individual in one of 14 demographic subpopulations will receive at least one false positive for one of these eleven diseases in a lifetime. We specify a suitable statistical model to account for the hierarchical structure of the data, and we use the parametric bootstrap to quantify the uncertainty surrounding our estimates. The estimated probability of receiving at least one false positive in a lifetime is 85.5% (±0.9%) and 38.9% (±3.6%) for baseline groups of women and men, respectively. It is higher for subpopulations recommended to screen more frequently than the baseline, including more vulnerable groups such as pregnant women and men who have sex with men. Since screening technology is imperfect, false positives remain inevitable. The high lifetime risk of a false positive reveals the importance of educating patients about this phenomenon.

## 1 Introduction

Healthcare professionals encourage individuals to get screened regularly for certain cancers, sexually transmitted diseases (STDs), and other medical conditions. Programs of repeated screening are beneficial because they allow for the early detection of these diseases, which, in turn, increases the likelihood of successful treatment. However, since even the most sophisticated screening technology falls short of 100% accuracy, some test results will inevitably be incorrect. Specifically, the result of any screening test might wind up being a false negative, indicating that the patient does not have the disease when they do, or a false positive, indicating that the patient has the disease when they do not.

Incorrect screening test results occur for a variety of reasons, ranging from improper specimen collection to transcription and reporting inaccuracies [1]. Regardless of their cause, screening test errors have serious implications. False negatives may delay the detection of potentially life-threatening diseases, reduce public confidence in screening, and, in some cases, induce legal action by the affected party [2]. False positives, on the other hand, take a toll on the recipient’s mental health, as they can generate stress and strain personal relationships [3–7]. There is also evidence that false positives reduce compliance with subsequent screenings [8, 9]. Further, false positives often require follow-up tests, and they may even prompt individuals to undergo unnecessary and costly invasive procedures [10].

Despite these adverse psychological, medical, and financial effects, efforts to quantify and communicate the risk of a false positive have remained scarce. For most recommended procedures, reliable data exist regarding the false positive rate of a single screening occasion. However, these rates are far from common knowledge among the general public. There have also been relatively few attempts to estimate the probability of receiving at least one false positive when a particular procedure is repeated over time [11–14]. Even less pursued is the estimation of this probability across multiple screening procedures for different diseases [15, 16]. Still absent in the literature is an effort to estimate the probability of receiving at least one false positive in a lifetime when an individual adheres to a program of repeated screening for several diseases.

In this manuscript, we estimate the lifetime probability of a false positive for individuals adhering to the screening guidelines of the United States Preventive Services Task Force (USPSTF) [17], which are widely considered the gold standard among medical practitioners. We consolidate the USPSTF recommendations and evidence into a data set that reports the results of 116 studies, each of which summarizes the accuracy of a screening procedure for one of five cancers or six STDs. We use these data to estimate the probability of receiving at least one false positive in a lifetime from any of the screening procedures considered, and we replicate our analysis for 14 demographic and behavioral subpopulations. Our findings provide patients and healthcare providers with an individualized layer of information that sheds new light on the long-term significance of screening test imprecision.

## 2 Methods

### 2.1 Diseases and screening procedures

Many different procedures are used to screen individuals for cancers, STDs, and other diseases like diabetes and osteoporosis. However, most procedures are recommended only for individuals at high risk for the disease, often only when risk factors emerge or intensify. Our analysis focuses on the set of diseases for which routine screening is advocated for large segments of the population. In addition, we consider only those diseases for which the negative consequences of false positives are well-documented or can be reasonably inferred based on the existing literature.

We formalize these principles by using the following criteria to identify the diseases relevant to our analysis: (1) The disease must be a cancer [18] or an STD [19] and (2) the USPSTF must have assigned a grade of C or higher to the screening service for the disease in its most recent recommendation—i.e., at minimum, it must recommend the service to be offered selectively to patients based on their unique circumstances. As detailed in S1 Table, there are five cancers (breast, cervical, colorectal, lung, and prostate) [20–24] and six STDs (chlamydia, gonorrhea, hepatitis B, hepatitis C, HIV, and syphilis) [25–31] that satisfy these criteria.

Some of these eleven diseases have only one recommended screening modality, while others have several. However, for nearly all diseases where multiple screening procedures are available, one is more common than the others in practice. With this in mind, we focus on only the primary screening procedure for each disease. The only two diseases for which the most common procedure is unclear are HIV and syphilis; for these, we select the primary screening modality based on the quantity and recency of available data.

### 2.2 Subpopulations and screening intervals

Not every individual is recommended to get screened for all eleven diseases listed above. For each cancer and STD, the USPSTF guidelines target only those segments of the population for which there are proven benefits to screening. These groups are defined according to demographic characteristics like age and sex or behavioral considerations like sexual activity and smoking status.

We account for this heterogeneity in screening protocols by replicating our analysis for 14 demographic and behavioral subpopulations. The six female subpopulations are defined according to the individual’s smoking status and the number of pregnancies they expect to experience in their lifetime. The eight male subpopulations are determined by the individual’s smoking status, whether or not they are a man who has sex with men (MSM), and whether or not they intend to get screened for prostate cancer, which is optional according to the USPSTF [24]. Baseline females are non-smokers who anticipate zero pregnancies in their lifetime, while baseline males are non-smoking, non-MSM individuals who do not intend to receive routine prostate exams. For both females and males, we define smoking status according to the USPSTF eligibility criteria for lung cancer screening—i.e., smokers are individuals with a 20 pack-year history who currently smoke or have quit within the past 15 years [23].

While other, more individualized risk factors—namely, one’s family history of cancer—certainly may influence screening practices on an individual basis, they have been omitted in the analysis presented here because they do not directly inform the highest-grade USPSTF recommendation for any of the eleven diseases considered. As such, we proceed using only the characteristics and behaviors listed in the previous paragraph, with the stipulation that the lifetime false positive probabilities reported below are conservative estimates that should be interpreted as lower bounds—i.e., the lifetime probability of a false positive is at least as high as our estimates.

**Fig 1:**
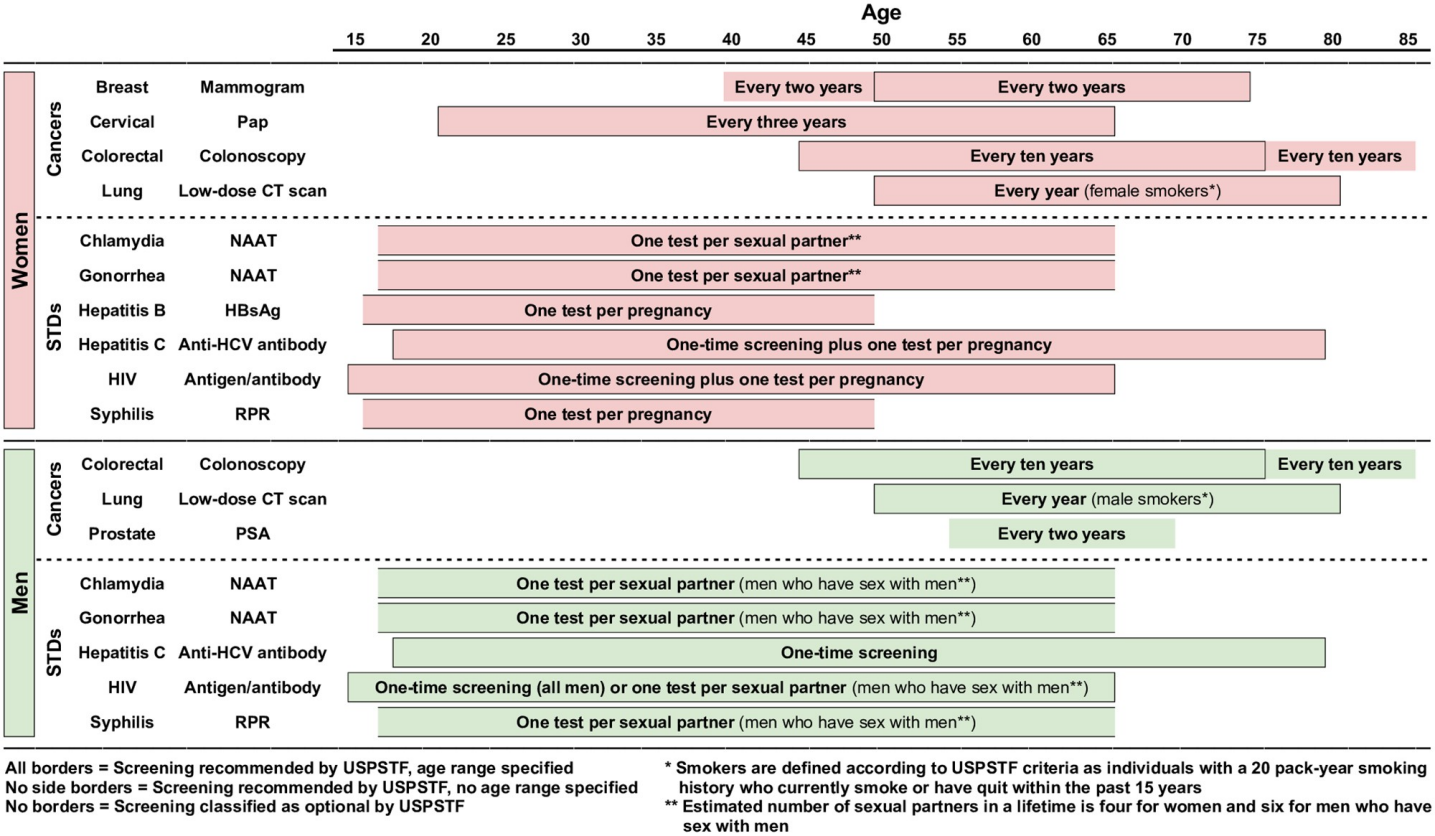
Timeline of screening guidelines.

Since the necessity and frequency of screening vary by subpopulation for each disease, so too does the number of times that individuals are recommended to get screened in a lifetime. For some diseases, the lifetime number of screening occasions follows clearly from the USPSTF guidelines. For others, the USPSTF guidelines lack either an age range, an interval at which screening should be repeated, or both, which makes it more complicated to derive the lifetime number of screening occasions for each subpopulation. S1 Appendix explains our approach to these more ambiguous cases, and S2 Table reports the assumed lifetime number of screening occasions for each disease by subpopulation. Fig 1 lists the primary screening procedure and summarizes the USPSTF guidelines for each disease considered in our analysis.

### 2.3 Data collection

Given the number of times an individual is recommended to get screened for a particular disease in a lifetime and provided that we have an estimate of the false positive rate for each screening occasion, we can estimate the probability that at least one of these occasions will result in a false positive. Let *p*_*d*_ denote the probability that an individual tests positive for disease *d* when the disease is not present. Our first objective is to obtain an estimate, ${\hat{p}}_{d}$, of *p*_*d*_.

To compile the data necessary to obtain ${\hat{p}}_{d}$, we first identified the most recent USPSTF recommendation statement for each disease as of August 31, 2021 (see S1 Table). Next, we extracted all sources among those cited by the USPSTF that reported the number of true positives, false negatives, true negatives, and false positives from a study involving the primary screening procedure for that disease. If no such studies were cited in the USPSTF recommendation statement for a particular disease, we referred to the corresponding USPSTF evidence review and proceeded similarly. In cases where neither of the two current documents contained relevant studies, we applied the same strategy to the next most recent set of USPSTF guidelines. See S3 Table for more details about the data collection procedure for each disease.

This process yielded 116 studies. Let *S*_*d*_ denote the number of studies collected for disease *d* using the above methodology. The estimated false positive rate of the primary screening procedure for disease *d* is given by

$$\begin{array}{c} {\hat{p}}_{d}=\frac{\sum_{s=1}^{S_{d}}{\text{FP}}_{s}}{\sum_{s=1}^{S_{d}}n_{s}}, \end{array}$$ (1)

where FP_*s*_ is the number of false positives in study *s* ∈ {1, …, *S*_*d*_} and *n*_*s*_ is the number of false positives plus the number of true negatives. It follows from (1) that ${\hat{p}}_{d}$ can be used to estimate the conditional probability of testing positive for disease *d* given that the disease is not present in the individual—i.e., the false positive probability of a single screening occasion for disease *d*. Table 1 reports the estimates obtained from (1) for each disease. Table 2 summarizes the notation used in Sections 2.3 and 2.4.

**Table 1:** Estimated false positive probability of one screening occasion.

Disease	Screening procedure	Estimate (SE)
Breast cancer	Mammogram	4.9% (0.1%)
Cervical cancer	Pap test	5.0% (0.1%)
Chlamydia	NAAT	0.5% (<0.1%)
Colorectal cancer	Colonoscopy	11.3% (1.3%)
Gonorrhea	NAAT	0.2% (<0.1%)
Hepatitis B	HBsAg test	2.0% (0.1%)
Hepatitis C	Anti-HCV antibody test	1.0% (0.2%)
HIV	Antigen/antibody test	0.2% (<0.1%)
Lung cancer	Low-dose CT scan	20.7% (0.1%)
Prostate cancer	PSA test	10.2% (0.3%)
Syphilis	RPR test	0.3% (<0.1%)

**Table 2:** Notation and definitions.

*S*_*d*_	Number of studies collected for disease *d*	*n*_*s*_	TN_*s*_ + FP_*s*_
TP_*s*_	Number of true positives observed in study *s*	*N*_*s*_	TP_*s*_ + FN_*s*_ + TN_*s*_ + FP_*s*_
FN_*s*_	Number of false negatives observed in study *s*	${\hat{p}}_{\text{FP},s}$	Proportion of cases in study *s* that are false positives
TN_*s*_	Number of true negatives observed in study *s*	${\hat{p}}_{\text{TN},s}$	Proportion of cases in study *s* that are true negatives
*FP*_*s*_	Number of false positives observed in study *s*	${\hat{p}}_{+,s}$	Proportion of cases in study *s* that are true positives or false negatives
*Z*_*s*_	Multinomial random variable representing the number of true positives, false negatives, true negatives, and false positives in study *s*		
*B*	Number of bootstrap iterations		
*p*_*d*_	Probability of receiving a false positive on one screening occasion for disease *d*		
*T*_*id*_	Number of times an individual in subpopulation *i* is recommended to get screened for disease *d* in a lifetime		
*P*_*id*_	Probability that a healthy individual in subpopulation *i* who adheres to the USPSTF screening guidelines will receive at least one false positive for disease *d* in a lifetime		
$D_{i}$	Set of diseases for which an individual in subpopulation *i* is recommended to get screened at least once in a lifetime		
*p*_*i*_	Probability that a healthy individual in subpopulation *i* who adheres to the USPSTF screening guidelines will receive at least one false positive for any of the diseases in $D_{i}$ in a lifetime		

### 2.4 Statistical methods

We seek to model the probability *p*_*i*_ that a healthy individual in subpopulation *i* will receive at least one false positive in a lifetime for at least one of the five cancers or six STDs considered, assuming that they adhere to the USPSTF screening guidelines. We define healthy individuals as those who remain negative for all five cancers and six STDs throughout their lifetime.

In order to model *p*_*i*_, we first model the probability *P*_*id*_ that a healthy individual in subpopulation *i* will receive at least one false positive for disease *d* in a lifetime, assuming that they get screened the recommended number of times *T*_*id*_ with the primary screening procedure for disease *d*. We also assume that, for each disease *d*, the results of each screening occasion are independent from the results of the previous occasions. Under these assumptions, the probability *P*_*id*_ can be expressed as

$$\begin{array}{c} P_{id}=1-{\left(1-p_{d}\right)}^{T_{id}}. \end{array}$$ (2)

Next, we extend this formula to incorporate more than one disease. Let $D_{i}$ denote the set of diseases for which an individual in subpopulation *i* is recommended to get screened at least once. We assume that the event of receiving at least one false positive in a lifetime for each disease $d\in D_{i}$ is independent from the same event for each of the other diseases in $D_{i}$. It follows from this assumption that the probability *p*_*i*_, as defined above, is given by

$$\begin{array}{c} p_{i}=1-\prod_{d\in D_{i}}\left(1-P_{id}\right)=1-\prod_{d\in D_{i}}{\left(1-p_{d}\right)}^{T_{id}}. \end{array}$$ (3)

The derivations of (2) and (3) are provided in S2 and S3 Appendices, respectively. These equations are informative in that they describe the relationship between *p*_*d*_, *P*_*id*_, and *p*_*i*_, but they are limited by the fact that these quantities are unknown in the real world. However, we can estimate (2) and (3) by plugging in the estimates ${\hat{p}}_{d}$ from (1). We obtain

$$\begin{array}{c} {\hat{P}}_{id}=1-{\left(1-{\hat{p}}_{d}\right)}^{T_{id}}\;\text{and}\;{\hat{p}}_{i}=1-\prod_{d\in D_{i}}\left(1-{\hat{P}}_{id}\right)=1-\prod_{d\in D_{i}}{\left(1-{\hat{p}}_{d}\right)}^{T_{id}}, \end{array}$$ (4)

where the latter can be used to estimate, as desired, the probability that a healthy individual in subpopulation *i* will receive at least one false positive in a lifetime.

Finally, to quantify the uncertainty surrounding our estimators, we rely on the assumption that the number of true positives (TP), false negatives (FN), true negatives (TN), and false positives (FP) from each study s can be modeled by a multinomial random variable $Z_{s}∼\text{Multinomial}\left(N_{s}\;,\;{\hat{p}}_{FP,s}\;,\;{\hat{p}}_{TN,s}\;,\;{\hat{p}}_{+,s}\right)$, where *N*_*s*_ is the total sample size of study *s*, ${\hat{p}}_{FP,s}$ is the proportion of cases in study *s* that are false positives, ${\hat{p}}_{TN,s}$ is the proportion that are true negatives, and ${\hat{p}}_{+,s}$ is the proportion that are true positives or false negatives. We incorporate the multinomial errors into our analysis by means of the parametric bootstrap [32]. Specifically, for each subpopulation *i*, we simulate from the $\text{Multinomial}(N_{s}\;,\;{\hat{p}}_{FP,s}\;,\;{\hat{p}}_{TN,s}\;,\;{\hat{p}}_{+,s})$ distribution to account for the inherent randomness of the results of each study *s* ∈ {1, …, *S*_*d*_} for each disease $d\in D_{i}$. We plug these simulated results into the equations for ${\hat{p}}_{d}$ and ${\hat{P}}_{id}$ to obtain one realization of ${\hat{p}}_{d}$ and ${\hat{P}}_{id}$ for each disease $d\in D_{i}$, as well as one realization of ${\hat{p}}_{i}$. We carry out this process *B* = 10, 000 times to obtain 10,000 realizations of each estimator, and we use these realizations to compute their respective standard errors. For ${\hat{p}}_{i}$, the standard error is given by

$$\begin{array}{c} SE\left({\hat{p}}_{i}\right)=\sqrt{\frac{1}{B-1}\sum_{b=1}^{B}{\left({\hat{p}}_{i}^{\;(b)}-{\bar{\hat{p}}}_{i}\right)}^{2}}, \end{array}$$ (5)

where ${\bar{\hat{p}}}_{i}=\frac{1}{B}\sum_{b=1}^{B}{\hat{p}}_{i}^{\;(b)}$. We use an analogous formula for SE(${\hat{p}}_{d}$) and SE(${\hat{P}}_{id}$). These standard errors are reported along with the corresponding point estimates.

## 3 Results

For each subpopulation *i*, Table 3 reports the estimated probability ${\hat{p}}_{i}$ that a healthy individual adhering to the USPSTF screening guidelines will receive at least one false positive in a lifetime for one of the five cancers or six STDs considered. S4 Table reports the lifetime probability of a false positive separately for the cancers and the STDs, and S5 Table breaks these probabilities down by disease for each subpopulation by reporting the estimates ${\hat{P}}_{id}$ from (4).

**Table 3:** Estimated lifetime false positive probability by subpopulation.

Subpopulation	Estimate (SE)
Baseline females	85.5% (0.9%)
Females, one pregnancy	86.0% (0.8%)
Females, two pregnancies	86.5% (0.8%)
Female smokers	88.5% (0.7%)
Female smokers, one pregnancy	88.9% (0.7%)
Female smokers, two pregnancies	89.3% (0.6%)
Baseline males	38.9% (3.6%)
Men who have sex with men (MSM)	43.1% (3.4%)
Male smokers	51.5% (2.9%)
MSM smokers	54.9% (2.7%)
Males, routine prostate exams	74.2% (1.7%)
MSM, routine prostate exams	76.0% (1.6%)
Male smokers, routine prostate exams	79.6% (1.3%)
MSM smokers, routine prostate exams	81.0% (1.2%)

The estimated lifetime probability of a false positive is at least 38% for all subpopulations and is greater than 50% for all but two. It exceeds 85% for all female subpopulations and is only slightly higher for female smokers (88.5% ± 0.7%) and pregnant women (86.0% ± 0.8%) than baseline females (85.5% ± 0.9%). There is far more variability in lifetime false positive risk among males than females, as the estimated lifetime probability of a false positive ranges from around 39% for baseline males (38.9% ± 3.6%) to more than 80% for MSM smokers who elect to get screened for prostate cancer (81.0% ± 1.2%).

We can use odds ratios to compare the lifetime risk of a false positive between subpopulations. The odds ratio for two subpopulations *i*_1_ and *i*_2_ is given by $\left({\hat{p}}_{i_{1}}/\left(1-{\hat{p}}_{i_{1}}\right)\right)/\left({\hat{p}}_{i_{2}}/\left(1-{\hat{p}}_{i_{2}}\right)\right)$, where ${\hat{p}}_{i_{1}}$ and ${\hat{p}}_{i_{2}}$ are the respective lifetime false positive probabilities for the two subpopulations.

Applying this formula to the estimates in Table 3, we find that the odds of receiving at least one false positive in a lifetime are 9.30 times higher for baseline females than baseline males and 7.27 times higher for female smokers than male smokers. Among females, the odds of receiving at least one false positive in a lifetime are 1.30 times higher for female smokers than baseline females, while they are 1.04 times higher for women who experience one pregnancy and 1.08 times higher for women who experience two pregnancies. These odds ratios are consistent with the small amount of variation in lifetime false positive risk among the female subpopulations. Among males, the odds of receiving at least one false positive in a lifetime are 1.19 times higher for men who have sex with men than baseline males, while they are 1.67 times higher for male smokers and 4.53 times higher for males who undergo routine prostate exams.

## 4 Discussion

To our knowledge, this manuscript is the first to quantify the lifetime risk of a false positive for individuals adhering to a program of repeated screening for multiple diseases. Our results indicate that healthy individuals who follow the USPSTF screening guidelines for a particular set of five cancers and six STDs have a high probability of receiving at least one false positive in a lifetime. However, this probability varies according to individuals’ demographic and behavioral characteristics. For instance, we estimate that the lifetime probability of a false positive is higher across the board for females than males, including more than twice as high for baseline females than baseline males. This discrepancy can be explained by the high lifetime false positive probability for breast cancer and cervical cancer, as women are recommended to get screened for these two diseases while men are not.

Similarly, we observe substantial variation in lifetime false positive risk among males, which is attributable to the fact that the USPSTF screening guidelines differ considerably across the eight male subpopulations. For instance, while baseline males are only recommended to get screened for one cancer and two STDs, MSM smokers who receive routine prostate exams are recommended to get screened for three cancers and five STDs (see S2 Table). Notably, the decision to get screened routinely for prostate cancer has a sizeable effect on lifetime false positive risk among males—the estimated lifetime false positive probability for men who receive routine prostate exams is nearly double that of baseline males. This is not surprising considering the high estimated probability of receiving a false positive from one prostate-specific antigen (PSA) test (see Table 1) and the relatively frequent time interval for prostate cancer screening (see Fig 1 and S2 Table).

Among females, on the other hand, we observe far less variability in our estimates of lifetime false positive risk. For instance, female smokers who experience two pregnancies have the highest risk out of the fourteen subpopulations considered, but the estimated lifetime false positive probability for these individuals is less than five percent higher than that of baseline females. This similarity between baseline and non-baseline females stems from the fact that women have a high probability of receiving at least one false positive in a lifetime for breast cancer and cervical cancer relative to the other cancers and STDs (see S5 Table), and the USPSTF guidelines for these two diseases are the same across all female subpopulations. The USPSTF guidelines for breast cancer and cervical cancer prescribe more screening occasions in a lifetime than the guidelines for the other cancers and STDs (see S2 Table), so it is not surprising that these two diseases contribute so prominently to lifetime false positive risk among females.

Our methodology relies on several assumptions about screening procedures, participants, and intervals. While our conclusions are robust to minor violations of these assumptions, we recognize that some of them may not hold in practice for all individuals. First, the probabilities estimated in this manuscript are conditional on the screening participant remaining negative for all five cancers and six STDs throughout their lifetime. The estimates reported in Tables 1 and 3, S4, and S5 Tables are not valid for individuals who contract one or more of the eleven diseases throughout their lifetime.

Second, the accuracy of our estimates depends on the extent to which patients and healthcare providers adhere to the USPSTF screening guidelines. We maintain that our assumption of strict adherence to the USPSTF guidelines is the most defensible way to determine the number of times an individual is recommended to get screened for a particular disease in a lifetime. Nevertheless, several studies suggest that the implementation of these guidelines in clinical settings is imperfect [33–35]. As such, the values reported in S2 Table may overestimate or underestimate the number of times individuals actually get screened for certain diseases in a lifetime.

Third, our assumption that individuals receive only the primary screening procedure for each disease likely does not capture the nuances of actual screening practices, which can vary across healthcare systems. Multiple screening procedures are available for several of the diseases considered in our analysis, and while one procedure tends to be more common than the others for each disease, the proportion of individuals that get screened with the other modalities may be nontrivial.

Finally, our determination of the lifetime number of screening occasions for the STDs relies on a few assumptions that may oversimplify the individualized nature of STD screening. The USPSTF guidelines for STD screening are highly contingent on risk factors and personal circumstances, and they generally do not provide both an age range and a time interval at which screening procedures should be repeated [25–31]. It is therefore possible, if not likely, that two individuals in the same subpopulation who both adhere to the USPSTF guidelines will get screened a different number of times for a particular STD in a lifetime. While it would be reasonable to estimate the lifetime number of STD screening occasions for each subpopulation empirically, the data required for this task are not readily available.

Even with these limitations in mind, our results offer undeniable evidence that the long-term burden of screening test imprecision is worth the attention of patients and healthcare providers. Specifically, our findings reveal the importance of communicating the pervasiveness of false positive results to patients, particularly more vulnerable individuals whose lifetime false positive risk is high relative to the baseline, such as pregnant women and men who have sex with men. This transparency is intended not to sow distrust in screening procedures or the guidelines associated with them, but rather to improve the ability of patients to respond sensibly to an unexpected positive result. An individual who is informed about the estimated lifetime false positive probability for someone with their demographic and behavioral characteristics is likely to exhibit a more measured response in this scenario than an uninformed individual.

To aid in this endeavor, we disseminate our findings via a web application called The False Positives Calculator [36]. This interactive tool, which is openly accessible at https://falsepositives.shinyapps.io/calculator, allows users to extract personalized information about screening test accuracy and learn about the USPSTF guidelines for the eleven diseases considered in our analysis. It also hosts our data set (free for download at https://github.com/timwhite0/false-positives-calculator), which, to the best of our knowledge, is the largest centralized, publicly available data source on screening test accuracy. More information about each of the 116 studies included in our analysis—such as the selection of subjects, sample size, exposures, and outcomes—can be obtained via the links provided in this data set.

False positive results are an inevitable feature of screening tests, as they cannot be eliminated without simultaneously increasing the prevalence of false negatives. For as long as screening technology remains imperfect, the best course of action for patients and healthcare providers is to remain informed about the risk of a false positive, paying particular attention to how this risk accumulates over time. This manuscript provides a framework for quantifying the lifetime risk of a false positive, one that could feasibly be updated as screening technology evolves and more data become available.

## Supporting information

S1 Table Inclusion criteria for each disease. (PDF)

S2 Table Lifetime number of screening occasions for each disease by subpopulation. (PDF)

S3 Table Data collection procedure for each disease. (PDF)

S4 Table Estimated lifetime false positive probability by subpopulation, cancers only and STDs only. (PDF)

S5 Table Estimated lifetime false positive probability for each disease by subpopulation. (PDF)

S1 Appendix Details about lifetime number of screening occasions. (PDF)

S2 Appendix Derivation of *P*_*id*_. (PDF)

S3 Appendix Derivation of *p*_*i*_. (PDF)
