# Supplementary material for: Estimating the lifetime risk of a false positive screening test result
Source: PLoS One. 2023 Feb 15;18(2):e0281153. doi: 10.1371/journal.pone.0281153 (PMC9931091; doi:10.1371/journal.pone.0281153)
Supplement: S1 Table — (PDF) [file pone.0281153.s001.pdf]

Estimating the lifetime risk of a false positive screening test result

Supporting information

Tim White and Sara Algeri

S1 Table: Inclusion criteria for each disease

Disease	Date of most recent USPSTF screening recommendation as of August 31, 2021	Cancer, STD, or neither	USPSTF grade of C or higher for at least some individuals	Included in analysis
Breast cancer	February 2016 [1]	Cancer [2]	Yes [1]	Yes
Cervical cancer	August 2018 [3]	Cancer [2]	Yes [3]	Yes
Chlamydia	December 2014 [4]	STD [5]	Yes [4]	Yes
Colorectal cancer	May 2021 [6]	Cancer [2]	Yes [6]	Yes
Gonorrhea	December 2014 [4]	STD [5]	Yes [4]	Yes
Hepatitis B	December 2020 [7] and July 2019 [8]	STD [5]	Yes [7, 8]	Yes
Hepatitis C	March 2020 [9]	STD [5]	Yes [9]	Yes
HIV	June 2019 [10]	STD [5]	Yes [10]	Yes
Lung cancer	March 2021 [11]	Cancer [2]	Yes [11]	Yes
Prostate cancer	May 2018 [12]	Cancer [2]	Yes [12]	Yes
Syphilis	June 2016 [13] and September 2018 [14]	STD [5]	Yes [13, 14]	Yes
Abdominal aortic aneurysm	December 2019 [15]	Neither [2, 5]	Yes [15]	No
Asymptomatic bacteriuria	September 2019 [16]	Neither [2, 5]	Yes [16]	No
Bladder cancer	August 2011 [17]	Cancer [2]	No [17]	No
Depression	March 2016 [18] and January 2016 [19]	Neither [2, 5]	Yes [18, 19]	No

Disease	Date of most recent USPSTF screening recommendation as of August 31, 2021	Cancer, STD, or neither	USPSTF grade of C or higher for at least some individuals	Included in analysis
Genital herpes	December 2016 [20]	STD [5]	No [20]	No
Gestational diabetes	August 2021 [21]	Neither [2, 5]	Yes [21]	No
Hypertension	April 2021 [22]	Neither [2, 5]	Yes [22]	No
Intimate partner violence, elder abuse, and abuse of vulnerable adults	October 2018 [23]	Neither [2, 5]	Yes [23]	No
Latent tuberculosis	September 2016 [24]	Neither [2, 5]	Yes [24]	No
Oral cancer	January 2014 [25]	Cancer [2]	No [25]	No
Osteoporosis	June 2018 [26]	Neither [2, 5]	Yes [26]	No
Ovarian cancer	February 2018 [27]	Cancer [2]	No [27]	No
Pancreatic cancer	August 2019 [28]	Cancer [2]	No [28]	No
Prediabetes and type 2 diabetes	August 2021 [29]	Neither [2, 5]	Yes [29]	No
Preeclampsia	April 2017 [30]	Neither [2, 5]	Yes [30]	No
Rh(D) incompatibility	February 2004 [31]	Neither [2, 5]	Yes [31]	No
Skin cancer	July 2016 [32]	Cancer [2]	No [32]	No
Testicular cancer	April 2011 [33]	Cancer [2]	No [33]	No
Thyroid cancer	May 2017 [34]	Cancer [2]	No [34]	No
Unhealthy drug use	June 2020 [35]	Neither [2, 5]	Yes [35]	No
Vision in children	September 2017 [36]	Neither [2, 5]	Yes [36]	No

Note: *The diseases listed in this table are those that satisfy one or both of the inclusion criteria defined in Section 2.1 of the manuscript — i.e., (1) the disease must be a cancer or an STD and (2) the USPSTF must have assigned a grade of C or higher to the screening service for the disease for at least some individuals.*

References

[1] Siu AL, U.S. Preventive Services Task Force. Screening for breast cancer: U.S. Preventive Services Task Force recommendation statement. Ann Intern Med. 2016; 164: 279-296.

[2] National Cancer Institute. Cancer Types. [cited 20 Nov 2022]. Available from: https://www.cancer.gov/types

[3] Curry SJ, Krist AH, Owens DK, Barry MJ, Caughey AB, Davidson KW, et al. Screening for cervical cancer: US Preventive Services Task Force recommendation statement. JAMA. 2018; 320: 674-686.

[4] LeFevre ML, U.S. Preventive Services Task Force. Screening for chlamydia and gonorrhea: U.S. Preventive Services Task Force recommendation statement. Ann Intern Med. 2014; 161: 902-910.

[5] Centers for Disease Control and Prevention. Diseases & Related Conditions. [cited 20 Nov 2022]. Available from: https://www.cdc.gov/std/general/default.htm

[6] Davidson KW, Barry MJ, Mangione CM, Cabana M, Caughey AB, Davis EM, et al. Screening for colorectal cancer: US Preventive Services Task Force recommendation statement. JAMA. 2021; 325: 1965-1977.

[7] Krist AH, Davidson KW, Mangione CM, Barry MJ, Cabana M, Caughey AB, et al. Screening for hepatitis B virus infection in adolescents and adults: US Preventive Services Task Force recommendation statement. JAMA. 2020; 324: 2415-2422.

[8] Owens DK, Davidson KW, Krist AH, Barry MJ, Cabana M, Caughey AB, et al. Screening for hepatitis B virus infection in pregnant women: US Preventive Services Task Force reaffirmation recommendation statement. JAMA. 2019; 322: 349-354.

[9] Owens DK, Davidson KW, Krist AH, Barry MJ, Cabana M, Caughey AB, et al. Screening for hepatitis C virus infection in adolescents and adults: US Preventive Services Task Force recommendation statement. JAMA. 2020; 323: 970-975.

[10] Owens DK, Davidson KW, Krist AH, Barry MJ, Cabana M, Caughey AB, et al. Screening for HIV infection: US Preventive Services Task Force recommendation statement. JAMA. 2019; 321: 2326-2336.

[11] Krist AH, Davidson KW, Mangione CM, Barry MJ, Cabana M, Caughey AB, et al. Screening for lung cancer: US Preventive Services Task Force recommendation statement. JAMA. 2021; 325: 962-970.

[12] Grossman DC, Curry SJ, Owens DK, Bibbins-Domingo K, Caughey AB, Davidson KW, et al. Screening for prostate cancer: US Preventive Services Task Force recommendation statement. JAMA. 2018; 319: 1901-1913.

[13] Bibbins-Domingo K, Grossman DC, Curry SJ, Davidson KW, Epling JW Jr, García FA, et al. Screening for syphilis infection in nonpregnant adults and adolescents: US Preventive Services Task Force recommendation statement. JAMA. 2016; 315: 2321-2327.

[14] Curry SJ, Krist AH, Owens DK, Barry MJ, Caughey AB, Davidson KW, et al. Screening for syphilis infection in pregnant women: US Preventive Services Task Force reaffirmation recommendation statement. JAMA. 2018; 320: 911-917.

[15] Owens DK, Davidson KW, Krist AH, Barry MJ, Cabana M, Caughey AB, et al. Screening for abdominal aortic aneurysm: US Preventive Services Task Force recommendation statement. JAMA. 2019; 322: 2211-2218.

[16] Owens DK, Davidson KW, Krist AH, Barry MJ, Cabana M, Caughey AB, et al. Screening for asymptomatic bacteriuria in adults: US Preventive Services Task Force recommendation statement. JAMA. 2019; 322: 1188-1194.

[17] Moyer VA, US Preventive Services Task Force. Screening for bladder cancer: US Preventive Services Task Force recommendation statement. Ann Intern Med. 2011;155: 246-251.

[18] Siu AL, U.S. Preventive Services Task Force. Screening for depression in children and adolescents: U.S. Preventive Services Task Force recommendation statement. Ann Intern Med. 2016; 164: 360-366.

[19] Siu AL, Bibbins-Domingo K, Grossman DC, Baumann LC, Davidson KW, Ebell M, et al. Screening for depression in adults: US Preventive Services Task Force recommendation statement. JAMA. 2016; 315: 380-387.

[20] Bibbins-Domingo K, Grossman DC, Curry SJ, Davidson KW, Epling JW Jr, García FA, et al. Serologic screening for genital herpes infection: US Preventive Services Task Force recommendation statement. JAMA. 2016; 316: 2525-2530.

[21] Davidson KW, Barry MJ, Mangione CM, Cabana M, Caughey AB, Davis EM, et al. Screening for gestational diabetes: US Preventive Services Task Force recommendation statement. JAMA. 2021; 326: 531-538.

[22] Krist AH, Davidson KW, Mangione CM, Cabana M, Caughey AB, Davis EM, et al. Screening for hypertension in adults: US Preventive Services Task Force reaffirmation recommendation statement. JAMA. 2021; 325: 1650-1656.

[23] Curry SJ, Krist AH, Owens DK, Barry MJ, Caughey AB, Davidson KW, et al. Screening for intimate partner violence, elder abuse, and abuse of vulnerable adults: US Preventive Services Task Force final recommendation statement. JAMA. 2018; 320: 1678-1687.

[24] Bibbins-Domingo K, Grossman DC, Curry SJ, Bauman L, Davidson KW, Epling JW Jr, et al. Screening for latent tuberculosis infection in adults: US Preventive Services Task Force recommendation statement. JAMA. 2016; 316: 962-969.

[25] Moyer VA, US Preventive Services Task Force. Screening for oral cancer: US Preventive Services Task Force recommendation statement. Ann Intern Med. 2014; 160: 55-60.

[26] Curry SJ, Krist AH, Owens DK, Barry MJ, Caughey AB, Davidson KW, et al. Screening for osteoporosis to prevent fractures: US Preventive Services Task Force recommendation statement. JAMA. 2018; 319: 2521-2531.

[27] Grossman DC, Curry SJ, Owens DK, Barry MJ, Davidson KW, Doubeni CA, et al. Screening for ovarian cancer: US Preventive Services Task Force recommendation statement. JAMA. 2018; 319: 588-594.

[28] Owens DK, Davidson KW, Krist AH, Barry MJ, Cabana M, Caughey AB, et al. Screening for pancreatic cancer: US Preventive Services Task Force reaffirmation recommendation statement. JAMA. 2019; 322: 438-444.

[29] Davidson KW, Barry MJ, Mangione CM, Cabana M, Caughey AB, Davis EM, et al. Screening for prediabetes and type 2 diabetes: US Preventive Services Task Force recommendation statement. JAMA. 2021; 326: 736-743.

[30] Bibbins-Domingo K, Grossman DC, Curry SJ, Barry MJ, Davidson KW, Doubeni CA, et al. Screening for preeclampsia: US Preventive Services Task Force recommendation statement. JAMA. 2017; 317: 1661-1667.

[31] US Preventive Services Task Force. Rh(D) incompatibility: Screening. 2004 [cited 20 Nov 2022]. Available from: https://www.uspreventiveservicestaskforce.org/uspstf/recommendation/rh-d-incompatibility-screening

[32] Bibbins-Domingo K, Grossman DC, Curry SJ, Davidson KW, Ebell M, Epling JW Jr, et al. Screening for skin cancer: US Preventive Services Task Force recommendation statement. JAMA. 2016; 316: 429-435.

[33] US Preventive Services Task Force. Screening for testicular cancer: US Preventive Services Task Force reaffirmation recommendation statement. Ann Intern Med. 2011; 154: 483-486.

[34] Bibbins-Domingo K, Grossman DC, Curry SJ, Barry MJ, Davidson KW, Doubeni CA, et al. Screening for thyroid cancer: US Preventive Services Task Force recommendation statement. JAMA. 2017; 317: 1882-1887.

[35] Krist AH, Davidson KW, Mangione CM, Barry MJ, Cabana M, Caughey AB, et al. Screening for unhealthy drug use: US Preventive Services Task Force recommendation statement. JAMA. 2020; 323: 2301-2309.

[36] Grossman DC, Curry SJ, Owens DK, Barry MJ, Davidson KW, Doubeni CA, et al. Vision screening in children aged 6 months to 5 years: US Preventive Services Task Force recommendation statement. JAMA. 2017; 318: 836-844.
