# Supplementary material for: Estimating the lifetime risk of a false positive screening test result
Source: PLoS One. 2023 Feb 15;18(2):e0281153. doi: 10.1371/journal.pone.0281153 (PMC9931091; doi:10.1371/journal.pone.0281153)
Supplement: S2 Table — (PDF) [file pone.0281153.s002.pdf]

Estimating the lifetime risk of a false positive screening test result

Supporting information

Tim White and Sara Algeri

S2 Table: Lifetime number of screening occasions for each disease by subpopulation

Female subpopulations

Disease	Screening procedure	FB	FP1	FP2	FS	FSP1	FSP2
Breast cancer	Mammogram	13	13	13	13	13	13
Cervical cancer	Pap test	15	15	15	15	15	15
Chlamydia	NAAT	4	4	4	4	4	4
Colorectal cancer	Colonoscopy	4	4	4	4	4	4
Gonorrhea	NAAT	4	4	4	4	4	4
Hepatitis B	HBsAg test	0	1	2	0	1	2
Hepatitis C	Anti-HCV antibody test	1	2	3	1	2	3
HIV	Antigen/antibody test	1	2	3	1	2	3
Lung cancer	Low-dose CT scan	0	0	0	1	1	1
Prostate cancer	PSA test	0	0	0	0	0	0
Syphilis	RPR test	0	1	2	0	1	2

Key:				
	FB	*Baseline females*	***FS***	*Female smokers*
	FP1	*Females, one pregnancy*	***FSP1***	*Female smokers, one pregnancy*
	FP2	*Females, two pregnancies*	***FSP2***	*Female smokers, two pregnancies*

Male subpopulations

**Disease**	**Screening procedure**	**MB**	**MSM**	**MS**	**MSMS**	**MP**	**MSMP**	**MPS**	**MSMPS**
Breast cancer	Mammogram	0	0	0	0	0	0	0	0
Cervical cancer	Pap test	0	0	0	0	0	0	0	0
Chlamydia	NAAT	0	6	0	6	0	6	0	6
Colorectal cancer	Colonoscopy	4	4	4	4	4	4	4	4
Gonorrhea	NAAT	0	6	0	6	0	6	0	6
Hepatitis B	HBsAg test	0	0	0	0	0	0	0	0
Hepatitis C	Anti-HCV antibody test	1	1	1	1	1	1	1	1
HIV	Antigen/antibody test	1	6	1	6	1	6	1	6
Lung cancer	Low-dose CT scan	0	0	1	1	0	0	1	1
Prostate cancer	PSA test	0	0	0	0	8	8	8	8
Syphilis	RPR test	0	6	0	6	0	6	0	6

Key:	***MB***	*Baseline males*	***MP***	*Males, routine prostate exams*
	MSM	*Men who have sex with men*	***MSMP***	*MSM, routine prostate exams*
	MS	*Male smokers*	***MPS***	*Male smokers, routine prostate exams*
	MSMS	*MSM smokers*	***MSMPS***	*MSM smokers, routine prostate exams*
