# Supplementary material for: Estimating the lifetime risk of a false positive screening test result
Source: PLoS One. 2023 Feb 15;18(2):e0281153. doi: 10.1371/journal.pone.0281153 (PMC9931091; doi:10.1371/journal.pone.0281153)
Supplement: S3 Table — (PDF) [file pone.0281153.s003.pdf]

Estimating the lifetime risk of a false positive screening test result

Supporting information

Tim White and Sara Algeri

S3 Table: Data collection procedure for each disease

Disease	Data collection procedure
Breast cancer	2016 USPSTF recommendation statement [1] → 2002 USPSTF evidence summary [2] → Mushlin et al 1998 [3] (**Malmo**, **Swedish Two-County**) → Baines et al 1988 [4] (**Canadian**), Frisell et al 1986 [5] (**Stockholm**)
Cervical cancer	2018 USPSTF recommendation statement [6] → 2018 USPSTF evidence review [7] (**ARTISTIC round 1**, **ARTISTIC round 2**, **FINNISH**, **NTCC phase I**, **NTCC phase II**, **POBASCAM round 1**, **POBASCAM round 2**, **SWEDESCREEN**)
Chlamydia	2014 USPSTF recommendation statement [8] → 2014 USPSTF evidence review [9] (**Chernesky 2005**, **Gaydos 2013**, **Schacter 2003**, **Schoeman 2012**, **Shrier 2004**, **Taylor 2011**, **Taylor 2012**, **Van Der Pol 2012a**)
Colorectal cancer	2021 USPSTF recommendation [10] → 2021 USPSTF evidence review [11] (**Zalis 2012**)
Gonorrhea	2014 USPSTF recommendation statement [8] → 2014 USPSTF evidence review [9] (**Chernesky 2005**, **Gaydos 2013**, **Stewart 2012**, **Taylor 2012**, **Van Der Pol 2012a**, **Van Der Pol 2012b**)
Hepatitis B	2020 USPSTF recommendation statement [12] → 2009 USPSTF reaffirmation recommendation statement [13] → 2004 USPSTF recommendation statement [14] → 2004 USPSTF evidence review [15] → 1996 USPSTF recommendation statement [16] → McCready et al 1991 [17] (**McCready 1991**), Toplikar et al 1993 [18] (**Toplikar 1993**)
Hepatitis C	2020 USPSTF recommendation statement [19] → 2013 USPSTF recommendation statement [20] → 2004 USPSTF recommendation statement [21] → 2004 USPSTF evidence review [22] (**Huber 1996**, **Prince 1997**) → Colin et al 2001 [23] (**Couroucé 1994**, **Janot 1994**, **Lavanchy 1996**, **Stuyver 1996**)
HIV	2019 USPSTF recommendation statement [24] → Branson et al 2014 [25] (**Bentsen 2011**, **Chavez 2011**, **Dubravac 2013**, **Masciotra 2011**, **Nasrullah 2013**, **Product insert 2010**, **Product insert 2011**)

**Disease**	**Data collection procedure**
Lung cancer	2021 USPSTF recommendation statement [26] → 2021 USPSTF evidence review [27] (**Becker 2015**, **De Koning 2020**, **Infante 2015**, **Lopes Pegna 2013**, **Pinsky 2013**, **Sverzellati 2016**)
Prostate cancer	2018 USPSTF recommendation statement [28] → 2018 USPSTF evidence review [29] → 2012 USPSTF recommendation statement [30] → 2011 USPSTF evidence review [31] → 2008 USPSTF recommendation statement [32] → Gann et al 1995 [33] (**Gann 1995**)<br><br>2008 USPSTF evidence update [34] → 2002 USPSTF recommendation statement [35] → 2002 USPSTF evidence update [36] → Mettlin et al 1996 [37] (**Mettlin 1996**), Jacobsen et al 1996 [38] (**Jacobsen 1996**)
Syphilis	2016 USPSTF recommendation statement (nonpregnant adults and adolescents) [39] → 2016 USPSTF evidence review (nonpregnant adults and adolescents) [40] → Ratnam 2005 [41] → Larsen et al 1998 [42] → Pettit et al 1983 [43] (**Pettit 1983**)<br><br>2018 USPSTF recommendation statement (pregnant women) [44] → 2018 USPSTF evidence review (pregnant women) [45] → Wang et al 2016 [46] (**Wang 2016**), Liu et al 2014 [47] (**Liu 2014**)

Note: *Bold text denotes the study IDs from the data set* [48].

References

[1] Siu AL, U.S. Preventive Services Task Force. Screening for breast cancer: U.S. Preventive Services Task Force recommendation statement. Ann Intern Med. 2016; 164: 279-296.

[2] Humphrey LL, Helfand M, Chan BKS, Woolf SH. Breast cancer screening: a summary of the evidence for the U.S. Preventive Services Task Force. Ann Intern Med. 2002; 137: 347-360.

[3] Mushlin AI, Kouides RW, Shapiro DE. Estimating the accuracy of screening mammography: a meta-analysis. Am J Prev Med. 1998; 14: 143-153.

[4] Baines CJ, McFarlane DV, Miller AB. Sensitivity and specificity of first screen mammography in 15 NBSS centres. Can Assoc Radiol J. 1988; 39: 273-276.

[5] Frisell J, Glas U, Hellstrom L, Somell A. Randomized mammographic screening for breast cancer in Stockholm. Breast Cancer Res Treat. 1986; 8: 45-54.

[6] Curry SJ, Krist AH, Owens DK, Barry MJ, Caughey AB, Davidson KW, et al. Screening for cervical cancer: US Preventive Services Task Force recommendation statement. JAMA. 2018; 320: 674-686.

[7] Melnikow J, Henderson JT, Burda BU, Senger CA, Durbin S, Soulsby MA. Screening for Cervical Cancer With High-Risk Human Papillomavirus Testing: A Systematic Evidence Review for the U.S. Preventive Services Task Force. Rockville, MD: Agency for Healthcare Research and Quality; 2018.

[8] LeFevre ML, U.S. Preventive Services Task Force. Screening for chlamydia and gonorrhea: U.S. Preventive Services Task Force recommendation statement. Ann Intern Med. 2014; 161: 902-910.

[9] Nelson HD, Zakher B, Cantor A, Deagas M, Pappas M. Screening for Gonorrhea and Chlamydia: Systematic Review to Update the U.S. Preventive Services Task Force Recommendations. Rockville, MD: Agency for Healthcare Research and Quality; 2014.

[10] Davidson KW, Barry MJ, Mangione CM, Cabana M, Caughey AB, Davis EM, et al. Screening for colorectal cancer: US Preventive Services Task Force recommendation statement. JAMA. 2021; 325: 1965-1977.

[11] Lin JS, Perdue LA, Henrikson NB, Bean SI, Blasi PR. Screening for Colorectal Cancer: An Evidence Update for the U.S. Preventive Services Task Force. Rockville, MD: Agency for Healthcare Research and Quality; 2021.

[12] Krist AH, Davidson KW, Mangione CM, Barry MJ, Cabana M, Caughey AB, et al. Screening for hepatitis B virus infection in adolescents and adults: US Preventive Services Task Force recommendation statement. JAMA. 2020; 324: 2415-2422.

[13] US Preventive Services Task Force. Screening for hepatitis B virus infection in pregnancy: U.S. Preventive Services Task Force reaffirmation recommendation statement. Ann Intern Med. 2009; 150: 869-873.

[14] US Preventive Services Task Force. Hepatitis B virus infection: Screening. 2004 [cited 20 Nov 2022]. Available from: https://www.uspreventiveservicestaskforce.org/uspstf/recommendation/hepatitis-b-virus-infection-screening-2004

[15] US Preventive Services Task Force. Final evidence review: Hepatitis B virus infection: Screening. 2004 [cited 20 Nov 2022]. Available from: https://www.uspreventiveservicestaskforce.org/uspstf/document/final-evidence-review46/hepatitis-b-virus-infection-screening-2004

[16] US Preventive Services Task Force. Hepatitis B virus infection: Screening, 1996. 1996 [cited 20 Nov 2022]. Available from: https://www.uspreventiveservicestaskforce.org/uspstf/recommendation/hepatitis-b-virus-infection-screening-1996

[17] McCready JA, Morens D, Fields HA, Coleman PJ, Kane M, Schatz G. Evaluation of enzyme immunoassay (EIA) as a screening method for hepatitis B markers in an open population. Epidemiol Infect. 1991; 107: 673-684.

[18] Toplikar E, Carlomagno A, Rojkin LF, Gariglio R, Lorenzo LE. Development of an enzyme immunoassay for the detection of hepatitis B surface antigen employing monoclonal antibodies. J Clin Lab Anal. 1993; 7: 324-328.

[19] Owens DK, Davidson KW, Krist AH, Barry MJ, Cabana M, Caughey AB, et al. Screening for hepatitis C virus infection in adolescents and adults: US Preventive Services Task Force recommendation statement. JAMA. 2020; 323: 970-975.

[20] Moyer VA. Screening for hepatitis C virus infection in adults: U.S. Preventive Services Task Force recommendation statement. Ann Intern Med. 2013; 159: 349-357.

[21] US Preventive Services Task Force. Hepatitis C virus infection: Screening, 2004. 2004 [cited 20 Nov 2022]. Available from: https://www.uspreventiveservicestaskforce.org/uspstf/recommendation/hepatitis-c-virus-infection-screening-2004

[22] Chou R, Clark EC, Helfand M. Screening for hepatitis C virus infection: a review of the evidence for the U.S. Preventive Services Task Force. Ann Intern Med. 2004; 140: 465-479.

[23] Colin C, Lanoir D, Touzet S, Meyaud-Kraemer L, Bailly F, Trepo C, et al. Sensitivity and specificity of third-generation hepatitis C virus antibody detection assays: an analysis of the literature. J Viral Hepat. 2001; 8: 87-95.

[24] Owens DK, Davidson KW, Krist AH, Barry MJ, Cabana M, Caughey AB, et al. Screening for HIV infection: US Preventive Services Task Force recommendation statement. JAMA. 2019; 321: 2326-2336.

[25] Branson BM, Owen SM, Wesolowski LG, Berry B, Werner BG, Wroblewski KE, et al. Laboratory testing for the diagnosis of HIV infection: updated recommendations. Centers for Disease Control and Prevention; 2014.

[26] Krist AH, Davidson KW, Mangione CM, Barry MJ, Cabana M, Caughey AB, et al. Screening for lung cancer: US Preventive Services Task Force recommendation statement. JAMA. 2021; 325: 962-970.

[27] Jonas DE, Reuland DS, Reddy SM, Nagle M, Clark SD, Weber RP, et al. Screening for Lung Cancer With Low-Dose Computed Tomography: An Evidence Review for the U.S. Preventive Services Task Force. Rockville, MD: Agency for Healthcare Research and Quality; 2021.

[28] Grossman DC, Curry SJ, Owens DK, Bibbins-Domingo K, Caughey AB, Davidson KW, et al. Screening for prostate cancer: US Preventive Services Task Force recommendation statement. JAMA. 2018; 319: 1901-1913.

[29] Fenton JJ, Weyrich MS, Durbin S, Liu Y, Bang H, Melnikow J. Prostate-Specific Antigen-Based Screening for Prostate Cancer: A Systematic Evidence Review for the U.S. Preventive Services Task Force. Rockville, MD: Agency for Healthcare Research and Quality; 2018.

[30] Moyer VA, U.S. Preventive Services Task Force. Screening for prostate cancer: U.S. Preventive Services Task Force recommendation statement. Ann Intern Med. 2012; 157: 120-134.

[31] Lin K, Croswell JM, Koenig H, Lam C, Maltz A. Prostate-Specific Antigen-Based Screening for Prostate Cancer: An Evidence Update for the U.S. Preventive Services Task Force. Rockville, MD: Agency for Healthcare Research and Quality; 2011.

[32] U.S. Preventive Services Task Force. Screening for prostate cancer: U.S. Preventive Services Task Force recommendation statement. Ann Intern Med. 2008; 149: 185-191.

[33] Gann PH, Hennekens CH, Stampfer MJ. A prospective evaluation of plasma prostate-specific antigen for detection of prostatic cancer. JAMA. 1995; 273: 289-294.

[34] Lin K, Lipsitz R, Miller T, Janakiraman S. Benefits and Harms of Prostate-Specific Antigen Screening for Prostate Cancer: An Evidence Update for the U.S. Preventive Services Task Force. Rockville, MD: Agency for Healthcare Research and Quality; 2008.

[35] US Preventive Services Task Force. Prostate cancer: Screening, 2002. 2002 [cited 20 Nov 2022]. Available from: https://www.uspreventiveservicestaskforce.org/uspstf/recommendation/prostate-cancer-screening-2002

[36] Harris R, Lohr KN. Screening for prostate cancer: an update of the evidence for the U.S. Preventive Services Task Force. Ann Intern Med. 2002; 137: 917-929.

[37] Mettlin C, Murphy GP, Babaian RJ, Chesley A, Kane RA, Littrup PJ, et al. The results of a five-year early prostate cancer detection intervention. Cancer. 1996; 77: 150-159.

[38] Jacobsen SJ, Bergstralh EJ, Guess HA, Katusic SK, Klee GG, Oesterling JE, et al. Predictive properties of serum-prostate-specific antigen testing in a community-based setting. Arch Intern Med. 1996; 156: 2462-2468.

[39] Bibbins-Domingo K, Grossman DC, Curry SJ, Davidson KW, Epling JW Jr, García FA, et al. Screening for syphilis infection in nonpregnant adults and adolescents: US Preventive Services Task Force recommendation statement. JAMA. 2016; 315: 2321-2327.

[40] Cantor A, Nelson HD, Daeges M, Pappas M. Screening for Syphilis in Nonpregnant Adolescents and Adults: Systematic Review to Update the 2004 U.S. Preventive Services Task Force Recommendation. Rockville, MD: Agency for Healthcare Research and Quality; 2016.

[41] Ratnam S. The laboratory diagnosis of syphilis. Can J Infect Dis Med Microbiol. 2005; 16: 45-51.

[42] Larsen SA, Pope V, Johnson RE, Kennedy EJ Jr. A Manual of Tests for Syphilis. Washington DC: American Public Health Association; 1998.

[43] Pettit DE, Larsen SA, Harbec PS, Feeley JC, Parham CE, Cruce DD, et al. Toluidine red unheated serum test, a nontreponemal test for syphilis. J Clin Microbiol. 1983; 18: 1141-1145.

[44] Curry SJ, Krist AH, Owens DK, Barry MJ, Caughey AB, Davidson KW, et al. Screening for syphilis infection in pregnant women: US Preventive Services Task Force reaffirmation recommendation statement. JAMA. 2018; 320: 911-917.

[45] Lin JS, Eder M, Bean S. Screening for Syphilis Infection in Pregnant Women: A Reaffirmation Evidence Update for the U.S. Preventive Services Task Force. Rockville, MD: Agency for Healthcare Research and Quality; 2018.

[46] Wang KD, Xu DJ, Su JR. Preferable procedure for the screening of syphilis in clinical laboratories in China. Infect Dis (Lond). 2016; 48: 26-31.

[47] Liu LL, Lin LR, Tong ML, Zhang HL, Huang SJ, Chen YY, et al. Incidence and risk factors for the prozone phenomenon in serologic testing for syphilis in a large cohort. Clin Infect Dis. 2014; 59: 384-389.

[48] White T. The False Positives Calculator. 2021 [cited 20 Nov 2022]. Available from: https://falsepositives.shinyapps.io/calculator
