# Supplementary material for: Estimating the lifetime risk of a false positive screening test result
Source: PLoS One. 2023 Feb 15;18(2):e0281153. doi: 10.1371/journal.pone.0281153 (PMC9931091; doi:10.1371/journal.pone.0281153)
Supplement: S4 Table — (PDF) [file pone.0281153.s004.pdf]

Estimating the lifetime risk of a false positive screening test result

Supporting information

Tim White and Sara Algeri

S4 Table: Estimated lifetime false positive probability by subpopulation, cancers only and STDs only

	Cancers only	***STDs only***
**Subpopulation**	**Estimate (SE)**	**Estimate (SE)**
Baseline females	85.0% (0.9%)	3.9% (0.2%)
Females, one pregnancy	85.0% (0.9%)	7.3% (0.4%)
Females, two pregnancies	85.0% (0.9%)	10.5% (0.5%)
Female smokers	88.1% (0.7%)	3.9% (0.2%)
Female smokers, one pregnancy	88.1% (0.7%)	7.3% (0.4%)
Female smokers, two pregnancies	88.1% (0.7%)	10.5% (0.5%)
Baseline males	38.2% (3.7%)	1.2% (0.2%)
Men who have sex with men (MSM)	38.2% (3.7%)	8.0% (0.3%)
Male smokers	50.9% (2.9%)	1.2% (0.2%)
MSM smokers	50.9% (2.9%)	8.0% (0.3%)
Males, routine prostate exams	73.9% (1.7%)	1.2% (0.2%)
MSM, routine prostate exams	73.9% (1.7%)	8.0% (0.3%)
Male smokers, routine prostate exams	79.3% (1.3%)	1.2% (0.2%)
MSM smokers, routine prostate exams	79.3% (1.3%)	8.0% (0.3%)
