# Supplementary material for: Estimating the lifetime risk of a false positive screening test result
Source: PLoS One. 2023 Feb 15;18(2):e0281153. doi: 10.1371/journal.pone.0281153 (PMC9931091; doi:10.1371/journal.pone.0281153)
Supplement: S5 Table — (PDF) [file pone.0281153.s005.pdf]

Estimating the lifetime risk of a false positive screening test result

Supporting information

Tim White and Sara Algeri

S5 Table: Estimated lifetime false positive probability for each disease by subpopulation

Female subpopulations

Disease	Screening procedure	FB	FP1	FP2	FS	FSP1	FSP2
Breast cancer	Mammogram	47.7% (0.4%)	47.7% (0.4%)	47.7% (0.4%)	47.7% (0.4%)	47.7% (0.4%)	47.7% (0.4%)
Cervical cancer	Pap test	53.5% (0.4%)	53.5% (0.4%)	53.5% (0.4%)	53.5% (0.4%)	53.5% (0.4%)	53.5% (0.4%)
Chlamydia	NAAT	2.0% (0.1%)	2.0% (0.1%)	2.0% (0.1%)	2.0% (0.1%)	2.0% (0.1%)	2.0% (0.1%)
Colorectal cancer	Colonoscopy	38.2% (3.7%)	38.2% (3.7%)	38.2% (3.7%)	38.2% (3.7%)	38.2% (3.7%)	38.2% (3.7%)
Gonorrhea	NAAT	0.8% (0.1%)	0.8% (0.1%)	0.8% (0.1%)	0.8% (0.1%)	0.8% (0.1%)	0.8% (0.1%)
Hepatitis B	HBsAg test	x	2.0% (0.1%)	4.1% (0.3%)	x	2.0% (0.1%)	4.1% (0.3%)
Hepatitis C	Anti-HCV antibody test	1.0% (0.2%)	1.9% (0.3%)	2.9% (0.5%)	1.0% (0.2%)	1.9% (0.3%)	2.9% (0.5%)
HIV	Antigen/antibody test	0.2% (<0.1%)	0.4% (<0.1%)	0.6% (0.1%)	0.2% (<0.1%)	0.4% (<0.1%)	0.6% (0.1%)
Lung cancer	Low-dose CT scan	x	x	x	20.7% (0.1%)	20.7% (0.1%)	20.7% (0.1%)
Prostate cancer	PSA test	x	x	x	x	x	x
Syphilis	RPR test	x	0.3% (<0.1%)	0.6% (0.1%)	x	0.3% (<0.1%)	0.6% (0.1%)

Key:	***FB***	*Baseline females*	***FS***	*Female smokers*
	FP1	*Females, one pregnancy*	***FSP1***	*Female smokers, one pregnancy*
	FP2	*Females, two pregnancies*	***FSP2***	*Female smokers, two pregnancies*

Male subpopulations

Disease	Screening procedure	MB	MSM	MS	MSMS	MP	MSMP	MPS	MSMPS
Breast cancer	Mammogram	x	x	x	x	x	x	x	x
Cervical cancer	Pap test	x	x	x	x	x	x	x	x
Chlamydia	NAAT	x	3.0% (0.2%)	x	3.0% (0.2%)	x	3.0% (0.2%)	x	3.0% (0.2%)
Colorectal cancer	Colonoscopy	38.2% (3.7%)	38.2% (3.7%)	38.2% (3.7%)	38.2% (3.7%)	38.2% (3.7%)	38.2% (3.7%)	38.2% (3.7%)	38.2% (3.7%)
Gonorrhea	NAAT	x	1.2% (0.2%)	x	1.2% (0.2%)	x	1.2% (0.2%)	x	1.2% (0.2%)
Hepatitis B	HBsAg test	x	x	x	x	x	x	x	x
Hepatitis C	Anti-HCV antibody test	1.0% (0.2%)	1.0% (0.2%)	1.0% (0.2%)	1.0% (0.2%)	1.0% (0.2%)	1.0% (0.2%)	1.0% (0.2%)	1.0% (0.2%)
HIV	Antigen/antibody test	0.2% (<0.1%)	1.2% (0.1%)	0.2% (<0.1%)	1.2% (0.1%)	0.2% (<0.1%)	1.2% (0.1%)	0.2% (<0.1%)	1.2% (0.1%)
Lung cancer	Low-dose CT scan	x	x	20.7% (0.1%)	20.7% (0.1%)	x	x	20.7% (0.1%)	20.7% (0.1%)
Prostate cancer	PSA test	x	x	x	x	57.9% (1.1%)	57.9% (1.1%)	57.9% (1.1%)	57.9% (1.1%)
Syphilis	RPR test	x	1.9% (0.2%)	x	1.9% (0.2%)	x	1.9% (0.2%)	x	1.9% (0.2%)

Key:				
	MB	*Baseline males*	***MP***	*Males, routine prostate exams*
	MSM	*Men who have sex with men*	***MSMP***	*MSM, routine prostate exams*
	MS	*Male smokers*	***MPS***	*Male smokers, routine prostate exams*
	MSMS	*MSM smokers*	***MSMPS***	*MSM smokers, routine prostate exams*
