# Supplementary material for: Estimating the lifetime risk of a false positive screening test result
Source: PLoS One. 2023 Feb 15;18(2):e0281153. doi: 10.1371/journal.pone.0281153 (PMC9931091; doi:10.1371/journal.pone.0281153)
Supplement: S1 Appendix — (PDF) [file pone.0281153.s006.pdf]

Estimating the lifetime risk of a false positive screening test result

Supporting information

Tim White and Sara Algeri

S1 Appendix: Details about lifetime number of screening occasions

The number of times that individuals are recommended to get screened in a lifetime varies by subpopulation for each disease. For some diseases, it is straightforward to derive the lifetime number of screening occasions from the USPSTF guidelines. This is the case for breast cancer, as biennial mammography for women aged 50 to 74 implies 13 mammograms in a lifetime for each female subpopulation [1]. The same is true for cervical cancer — a woman who gets screened with a Pap test every three years between the ages of 21 and 65 will receive 15 Pap tests in a lifetime [2]. It is also true for colorectal cancer, as an individual who gets a colonoscopy every ten years between the ages of 45 and 75 will receive a lifetime total of four colonoscopies [3].

For other diseases, the USPSTF guidelines lack either an age range, an interval at which screening should be repeated, or both. In these ambiguous cases, we adopt a conservative approach by imposing assumptions that are more likely to underestimate, rather than overestimate, the lifetime number of screening occasions for a particular subpopulation. This approach ensures that our estimates do not overstate the lifetime risk of a false positive. In fact, for the cases described below where we adopt this conservative approach, our estimates should be interpreted as lower bounds — i.e., the lifetime probability of a false positive for these diseases is at least as high as our estimates.

Unlike the USPSTF cancer screening guidelines, the USPSTF guidelines for the STDs do not specify an age range or an interval at which screening should be repeated. Rather, they suggest that screening should be contingent on new or persistent risk factors or that, in the cases of HIV [4] and hepatitis C [5], repeated screening is not necessary for most individuals. We eliminate this ambiguity for several STDs by imposing the assumption that females and men who have sex with men receive one test per sexual partner, of which we assume there are four [6] for females and six [7] for MSM in a lifetime, on average. This assumption is not relevant for the non-MSM male subpopulations because these individuals are not presumed to be at

increased risk for any of the STDs considered.

Another complication arising from the STD screening guidelines is that the USPSTF and CDC [8] recommend additional tests during each pregnancy for most STDs, usually at the first prenatal visit (although high-risk pregnant women may benefit from repeated screening closer to delivery). The exceptions to this are chlamydia and gonorrhea, for which the USPSTF finds no evidence of substantial net benefits from screening average-risk pregnant women [9] and the CDC only advocates additional tests for average-risk pregnant women under 25 [8], which is younger than the mean age of pregnancy in the United States [10]. As such, we assume one additional screening occasion per pregnancy for hepatitis B, hepatitis C, HIV, and syphilis and no additional screening occasions per pregnancy for chlamydia and gonorrhea.

Finally, we employ our conservative approach when determining the number of screening occasions recommended in a lifetime for lung cancer and prostate cancer. For lung cancer, the USPSTF recommends annual screening with low-dose computed tomography for individuals between the ages of 50 and 80 who have a 20 pack-year history and who either currently smoke or have quit within the past 15 years [11]; this implies 31 screening occasions in a lifetime for eligible individuals. However, since the estimated false positive rate of each low-dose CT scan is more than 20% (see Table 1 in the manuscript), a healthy individual is almost certain to receive at least one false positive from 31 low-dose CT scans. In turn, the probability that a smoker will receive at least one false positive from any screening procedure in a lifetime also approaches 100%. Because of this, and because lung cancer screening is recognized as a service with low uptake [11], we instead assume that male and female smokers receive only one low-dose CT scan in a lifetime. For prostate cancer, the USPSTF endorses optional screening for males between the ages of 55 and 69, but notes that there is limited evidence regarding the optimal screening interval for this service [12]. We assume that males who elect to get screened for prostate cancer do so every two years, as recommended by the American Cancer Society [13] and the American Urological Association [14].

References

[1] Siu AL, U.S. Preventive Services Task Force. Screening for breast cancer: U.S. Preventive Services Task Force recommendation statement. Ann Intern Med. 2016; 164: 279-296.

[2] Curry SJ, Krist AH, Owens DK, Barry MJ, Caughey AB, Davidson KW, et al. Screening for cervical cancer: US Preventive Services Task Force recommendation statement. JAMA. 2018; 320: 674-686.

[3] Davidson KW, Barry MJ, Mangione CM, Cabana M, Caughey AB, Davis EM, et al. Screening for colorectal cancer: US Preventive Services Task Force recommendation statement. JAMA. 2021; 325: 1965-1977.

[4] Owens DK, Davidson KW, Krist AH, Barry MJ, Cabana M, Caughey AB, et al. Screening for HIV infection: US Preventive Services Task Force recommendation statement. JAMA. 2019; 321: 2326-2336.

[5] Owens DK, Davidson KW, Krist AH, Barry MJ, Cabana M, Caughey AB, et al. Screening for hepatitis C virus infection in adolescents and adults: US Preventive Services Task Force recommendation statement. JAMA. 2020; 323: 970-975.

[6] Centers for Disease Control and Prevention. Key Statistics from the National Survey of Family Growth - N Listing. [cited 20 Nov 2022]. Available from: https://www.cdc.gov/nchs/nsfg/key_statistics/n.htm

[7] Mercer CH, Prah P, Field N, Tanton C, Macdowall W, Clifton S, et al. The health and well-being of men who have sex with men (MSM) in Britain: Evidence from the third National Survey of Sexual Attitudes and Lifestyles (Natsal-3). BMC Public Health. 2016; 16: 1-16.

[8] Centers for Disease Control and Prevention. Recommended Clinician Timeline for Screening for Syphilis, HIV, HBV, HCV, Chlamydia, and Gonorrhea. [cited 20 Nov 2022]. Available from: https://www.cdc.gov/nchhstp/pregnancy/screening/clinician-timeline.html

[9] LeFevre ML, U.S. Preventive Services Task Force. Screening for chlamydia and gonorrhea: U.S. Preventive Services Task Force recommendation statement. Ann Intern Med. 2014; 161: 902-910.

[10] Organisation for Economic Co-operation and Development. SF2.3: Age of mothers at childbirth and age-specific fertility. 2021 [cited 20 Nov 2022]. Available from: https://www.oecd.org/els/soc/SF_2_3_Age_mothers_childbirth.pdf

[11] Krist AH, Davidson KW, Mangione CM, Barry MJ, Cabana M, Caughey AB, et al. Screening for lung cancer: US Preventive Services Task Force recommendation statement. JAMA. 2021; 325: 962-970.

[12] Grossman DC, Curry SJ, Owens DK, Bibbins-Domingo K, Caughey AB, Davidson KW, et al. Screening for prostate cancer: US Preventive Services Task Force recommendation statement. JAMA. 2018; 319: 1901-1913.

[13] American Cancer Society. American Cancer Society Recommendations for Prostate Cancer Early Detection. [cited 20 Nov 2022]. Available from: https://www.cancer.org/cancer/prostate-cancer/detection-diagnosis-staging/acs-recommendations.html

[14] Carter HB, Albertsen PC, Barry MJ, Etzioni R, Freedland SJ, Greene KL, et al. Early detection of prostate cancer: AUA Guideline. J Urol. 2013; 190: 419-426.
