# Supplementary material for: Estimating the lifetime risk of a false positive screening test result
Source: PLoS One. 2023 Feb 15;18(2):e0281153. doi: 10.1371/journal.pone.0281153 (PMC9931091; doi:10.1371/journal.pone.0281153)
Supplement: S2 Appendix — (PDF) [file pone.0281153.s007.pdf]

Estimating the lifetime risk of a false positive screening test result

Supporting information

Tim White and Sara Algeri

S2 Appendix: Derivation of P_{id}

Suppose a healthy individual in subpopulation i gets screened the recommended number of times T_{id} for disease d in their lifetime. We aim to derive the probability P_{id} that this individual will receive at least one false positive for disease d in their lifetime.

For all $j \in \{1, 2, ..., T_{id}\}$, let A_j denote the event where the individual receives a false positive the jth time they get screened for disease d. Note that P_{id} can be thought of as the probability that at least one of $A_1, A_2, ..., A_{T_{id}}$ occurs. Therefore:

$$P_{id} = P(A_1 \cup A_2 \cup ... \cup A_{T_{id}}) = 1 - (P(A_1 \cup A_2 \cup ... \cup A_{T_{id}}))^c$$

By De Morgan's law, we have:

$$... = 1 - P(A_1^c \cap A_2^c \cap ... \cap A_{T_{id}}^c)$$

By our assumption that the results of the T_{id} screening occasions are independent (see Section 2.4 of the manuscript), we have:

$$... = 1 - P(A_1^c) \cdot P(A_2^c) \cdot ... \cdot P(A_{T_{id}}^c) = 1 - (1 - P(A_1)) \cdot (1 - P(A_2)) \cdot ... \cdot (1 - P(A_{T_{id}}))$$

Recall from Section 2.3 that p_d denotes the probability that a healthy individual will receive a false positive for disease d from one screening occasion. It follows that for all $j \in \{1, 2, ..., T_{id}\}$, $P(A_j) = p_d$. Therefore:

$$... = 1 - (1 - p_d) \cdot (1 - p_d) \cdot ... \cdot (1 - p_d) = 1 - (1 - p_d)^{T_{id}}$$

Thus, we arrive at equation (2) from Section 2.4:

$$\boxed{P_{id} = 1 - (1 - p_d)^{T_{id}}}$$
