# Supplementary material for: Estimating the lifetime risk of a false positive screening test result
Source: PLoS One. 2023 Feb 15;18(2):e0281153. doi: 10.1371/journal.pone.0281153 (PMC9931091; doi:10.1371/journal.pone.0281153)
Supplement: S3 Appendix — (PDF) [file pone.0281153.s008.pdf]

Estimating the lifetime risk of a false positive screening test result

Supporting information

Tim White and Sara Algeri

S3 Appendix: Derivation of p_i

Recall from Section 2.4 of the manuscript that $\mathcal{D}_i$ denotes the set of diseases for which an individual in subpopulation i is recommended to get screened at least once. Let $|\mathcal{D}_i|$ denote the size of the set $\mathcal{D}_i$. Suppose a healthy individual in subpopulation i gets screened the recommended number of times in their lifetime for all diseases in $\mathcal{D}_i$. We seek to derive the probability p_i that this individual will receive at least one false positive for at least one of the diseases in $\mathcal{D}_i$ in their lifetime.

For all $k \in \{1, 2, ..., |\mathcal{D}_i|\}$, let B_k denote the event where the individual receives at least one false positive in a lifetime for the kth disease in $\mathcal{D}_i$. Note that p_i can be thought of as the probability that at least one of $B_1, B_2, ..., B_{|\mathcal{D}_i|}$ occurs. Therefore:

$$p_i = P(B_1 \cup B_2 \cup ... \cup B_{|\mathcal{D}_i|}) = 1 - (P(B_1 \cup B_2 \cup ... \cup B_{|\mathcal{D}_i|}))^c$$

By De Morgan's law, we have:

$$... = 1 - P(B_1^c \cap B_2^c \cap ... \cap B_{|\mathcal{D}_i|}^c)$$

Recall our assumption from Section 2.4 that the event of receiving at least one false positive in a lifetime for each disease in $\mathcal{D}_i$ is independent from the same event for each of the other diseases in $\mathcal{D}_i$. Therefore:

$$... = 1 - P(B_1^c) \cdot P(B_2^c) \cdot ... \cdot P(B_{|\mathcal{D}_i|}^c) = 1 - (1 - P(B_1)) \cdot (1 - P(B_2)) \cdot ... \cdot (1 - P(B_{|\mathcal{D}_i|}))$$

Recall also that P_{id} denotes the probability that a healthy individual in subpopulation i will receive at least one false positive in a lifetime for some disease $d \in \mathcal{D}_i$. Let $d_1, d_2, ..., d_{|\mathcal{D}_i|}$ denote the diseases in $\mathcal{D}_i$. It follows that for all $k \in \{1, 2, ..., |\mathcal{D}_i|\}$, $P(B_k) = P_{id_k}$. Therefore:

$$... = 1 - (1 - P_{id_1}) \cdot (1 - P_{id_2}) \cdot ... \cdot (1 - P_{id_{|\mathcal{D}_i|}}) = 1 - \prod_{d \in \mathcal{D}_i} (1 - P_{id})$$

Thus, we arrive at equation (3) from Section 2.4. We can plug in $P_{id} = 1 - (1 - p_d)^{T_{id}}$ to obtain the full expression presented in Section 2.4:

$$\boxed{p_i = 1 - \prod_{d \in \mathcal{D}_i} (1 - P_{id}) = 1 - \prod_{d \in \mathcal{D}_i} (1 - p_d)^{T_{id}}}$$
